# Potential Drug Interactions in Psychiatric Patients Undergoing Pangenotypic Therapy for Hepatitis C Virus Infection

**DOI:** 10.3390/ph19010087

**Published:** 2026-01-01

**Authors:** Dorota Dybowska, Małgorzata Pawłowska, Dorota Kozielewicz

**Affiliations:** Department of Infectious Diseases and Hepatology, Faculty of Medicine, Collegium Medicum in Bydgoszcz, Nicolaus Copernicus University, 87-100 Toruń, Poland

**Keywords:** direct-acting antivirals, hepatitis C virus, drug–drug interactions, mental disorders, psychotropic drugs

## Abstract

Over the past decade, significant progress has been made in the treatment of chronic hepatitis C virus (HCV) infection. The introduction of direct-acting antivirals (DAAs) has revolutionized the treatment of HCV infection, offering nearly 100% efficacy. Furthermore, additional therapeutic regimens with pangenotypic efficacy have been registered. These drugs are also characterized by a few adverse events and good treatment tolerance. As DAA therapy is now accessible to virtually all patients, including those with multimorbidity who often take multiple medications, drug interactions (DDIs) have become a significant clinical challenge. One of the groups of patients who are frequently infected with HCV is those with mental disorders. Due to frequently overlapping metabolic pathways, DDIs can occur, affecting the effectiveness of both psychiatric and antiviral therapy. Knowledge of these interactions is crucial in these cases and influences patient management. This paper discusses the most significant interactions between pangenotypic DAA regimens and psychotropic medications.

## 1. Introduction

The introduction of direct-acting anti-HCV (DAA) drugs has revolutionized the treatment of hepatitis C virus (HCV) infection. Sustained virological response rates are close to 100%, regardless of genotype, liver disease progression, patient age, gender, or previous ineffective non-DAA therapeutic regimen. The latest advancement in antiviral therapy is the approval of three pangenotypic treatment regimens: a combination of the protease inhibitor glecaprevir (GLE) with the nonstructural protein 5A (NS5A) inhibitor pibrentasvir (PIB); a polymerase inhibitor sofosbuvir (SOF) with velpatasvir (VEL), which works by inhibiting HCV NS5A; and a regimen using SOF, VEL, and the protease inhibitor voxilaprevir (VOX). The introduction of these drugs simplified therapy by eliminating the need for HCV genotyping [1]. These therapies are well tolerated with rare adverse events. However, it should be noted that these drugs affect the function of cytochrome P450 (CYP) and transport proteins, acting as inducers, inhibitors, or substrates. This effect can lead to drug interactions (DDIs), which every physician should pay attention to when preparing a patient for DAA therapy and during its implementation. This is important when treating patients with multiple comorbidities and consequent polypharmacy. A large group of HCV-infected patients are individuals with mental disorders. The incidence of HCV infection is estimated to be 3 to 20 times higher among patients with depression, schizophrenia, bipolar disorder, and personality disorders compared to the general population. This figure rises to 60–70% in individuals with a history of intravenous psychoactive substance use [2,3]. The occurrence of mental illnesses is associated with the need for psychotropic medication. In the Patel study, 16% of HCV-infected patients were taking antidepressants, and 10% were taking antipsychotics (n = 3716) [4]. In a Dutch study, benzodiazepines and selective serotonin reuptake inhibitors (SSRIs) were among the most frequently used drugs by HCV-infected patients [5]. It should be noted that both DAAs and psychoactive substances are metabolized in the liver and can affect the activity of cytochrome P450 and P-glycoprotein (P-gp). This can lead to drug interactions, resulting in adverse effects and/or treatment failure [6,7].

The aim of this paper is to discuss drug interactions between pangenotypic DAA regimens and medications used in the treatment of patients with mental disorders.

## 2. Methods

The PubMed database was searched for publications regarding drug–drug interactions (DDIs) between pangenotypic direct-acting antivirals (DAAs) used in HCV therapy and psychotropic medications, covering literature published up to 2025. The keywords used were “direct-acting antivirals,” “drug–drug interactions,” “pangenotypic regimens,” “mental disorders,” and “psychotropic drugs.” The Google search engine was used to identify articles and conference abstracts. Generic and/or brand names of medications were included in the search results.

The analysis included retrospective and prospective studies, review studies, and other like Editorials and Recommendations, and publications describing drug metabolic pathways. Articles written in languages other than English were excluded.

To verify the safety profile, each substance was cross-referenced with the University of Liverpool’s HEP Drug Interactions Checker. Interactions were categorized as follows: no interaction expected, potential weak interaction, potential interaction requiring monitoring, or contraindicated. Data from the European Association for the Study of the Liver recommendations on the treatment of hepatitis C were also included.

Additionally, the Summary of Product Characteristics published by the European Medicines Agency (EMA) for products containing glecaprevir/pibrentasvir, velpatasvir/sofosbuvir, and voxilaprevir/velpatasvir/sofosbuvir were reviewed, along with available descriptions of psychotropic drugs on the EMA, Food and Drug Administration, Health Products Regulatory Authority, Electronic Medicines Compendium, DrugBank, and ClinPgx platforms.

Initially, 126 records were identified, of which 80 were qualified for full analysis after removing duplicates, removing articles in other languages, and analyzing titles/abstracts. Ultimately, 43 publications and 25 Summary of Product Characteristics containing information on the metabolic pathways of the discussed drugs were included in the review.

## 3. Overview

Co-administration of drugs may alter their therapeutic effects. Interactions between substances often lead to a reduction or loss of efficacy of one of the therapeutic agents. Therapeutic failure due to DDIs occurs in 8.6–11.6% of cases, according to some studies [8]. The best characterized mechanisms of inhibition and induction are those related to cytochrome P450 isoenzymes, particularly CYP3A4, which is responsible for the oxidation of many drugs. The clinical impact of enzyme inhibition and induction depends, among other factors, on the broad therapeutic index of the drugs used. For example, a drug with a broad therapeutic index is less susceptible to the effects of an enzyme inhibitor/inducer despite altered serum concentrations of the active substance [9,10]. Benzodiazepines, tricyclic antidepressants, and antipsychotics have a narrow therapeutic index. In contrast, SSRIs and DAAs have a wide therapeutic range, making them less susceptible to concentration changes caused by, for example, CYP inhibition or induction [11]. In the case of polypharmacy, many drugs are substrates (S), inducers, and inhibitors (I) of the same CYP enzyme, which can lead to multidrug interactions [10].

**Figure 1:**
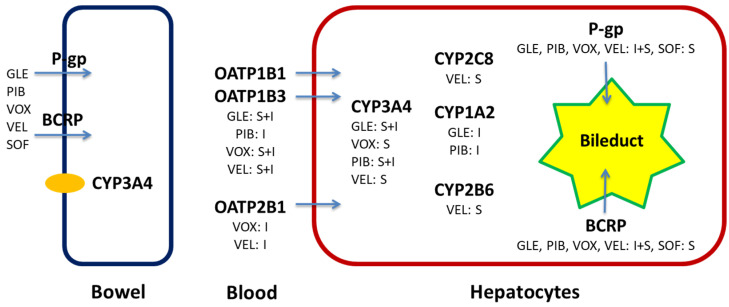
Drug metabolism enzymes and transporters involved in the metabolism and distribution of direct-acting antivirals. BCRP—breast cancer resistance protein; CYP—cytochrome; GLE—glecaprevir; I—inhibitor; OATP—organic anion-transporting polypeptide; PIB—pibrentasvir; P-gp—P-glycoprotein; SOF—sofosbuvir; S—substrate; VEL—velpatasvir; VOX—voxilaprevir.

Drug interactions can also be caused by transporter proteins that influence the absorption, distribution, or elimination of a drug. These include, for example, P-glycoprotein, organic anion-transporting polypeptide (OATP), and breast cancer resistance protein (BCRP). These proteins are present in enterocytes and hepatocytes, and in renal tubular epithelial cells, the blood–brain barrier, and the placenta. These transporters are subject to inhibition or induction, which can consequently lead to drug interactions [12,13]. Inhibition of P-gp in enterocytes increases the bioavailability of an orally administered drug, whereas its inhibition in the liver or kidneys results in decreased excretion. Inhibition of OATP1B1 can lead to increased drug concentrations by interfering with hepatic uptake, which affects the amount of therapeutic drug entering hepatocytes, the site of the main metabolic pathways. Direct-acting antivirals used to treat HCV infection are metabolized through these pathways, making them subject to drug interactions [14,15]. Figure 1 shows the metabolic scheme of DAAs used in pangenotypic regimens [16,17,18,19,20].

It is important to remember that many drugs simultaneously affect more than one target, leading to DDIs. For example, they can be substrates, inhibitors, or inducers of P-gp along with CYP 3A4 [21]. The most important mechanisms underlying DDIs between DAAs and psychoactive substances occur via hepatic metabolism and drug transporters [5].

### 3.1. Metabolic Pathways of Pangenotypic DAA Regimens

One of the direct-acting anti-HCV drugs with the lowest potential for DDIs is the NS5B inhibitor sofosbuvir. However, the potential for drug interactions among NS3/4A protease inhibitors in the treatment of HCV infection varies. Because most of them are both substrates and inhibitors of drug transporters and CYP enzymes, they appear to cause more DDIs than NS5A and NS5B inhibitors. The newest NS3/4A protease inhibitors, glecaprevir and voxilaprevir, have significantly fewer drug interactions than the first-introduced drugs [22,23,24,25,26].

#### 3.1.1. Glecaprevir/Pibrentasvir

Glecaprevir, an NS3/4A protease inhibitor, and pibrentasvir (PIB), an NS5A replication complex inhibitor, are used at a fixed dose to treat HCV genotype 1-6 infection.

##### Effect of Glecaprevir and Pibrentasvir on the Metabolism of Other Drugs

Glecaprevir and pibrentasvir are inhibitors of P-gp, breast cancer resistance protein, and OATP1B1/3. Co-administration with substances that are substrates of these proteins may increase plasma concentrations of these drugs (e.g., lurasidone). Some DDIs are significant enough to contraindicate their combination. In other situations, dose adjustments for substances that are substrates of P-gp, BCRP, and OATP should be considered, particularly in cases with a narrow therapeutic index [16,27,28].

Because GLE/PIB are weak CYP3A inhibitors, no clinically significant effect on increased concentrations of CYP3A substrates (e.g., midazolam or risperidone) has been observed. However, caution should be exercised when using higher doses of quetiapine, as this may require additional monitoring and appropriate action [16,24,25,27,28].

##### Effects of Other Drugs on Glecaprevir and Pibrentasvir

Glecaprevir is partially metabolized primarily by CYP3A4. Additionally, PIB and GLE are substrates of the P-gp and BRCP transporters, and glecaprevir is also a substrate of OATP1B1/3 [16].

Combining medicinal products that are strong inducers of P-gp and CYP3A, such as carbamazepine, phenytoin, primidone, phenobarbital, or St. John’s wort (Hypericum perforatum), with glecaprevir and/or pibrentasvir is contraindicated. They may significantly impact the therapeutic effects of these DAAs by reducing plasma GLE and/or PIB concentrations. Drugs that are moderate inducers of P-glycoprotein and CYP3A (e.g., oxcarbazepine) may also affect the concentration of glecaprevir and pibrentasvir; therefore, their co-administration is not recommended [16,27].

#### 3.1.2. Velpatasvir/Sofosbuvir

Velpatasvir (VEL), an inhibitor of the NS5A replication complex, along with sofosbuvir, an inhibitor of the NS5B nucleotide polymerase analogue, is another pangenotypic DAA regimen approved for the treatment of HCV infection.

SOF is a prodrug that is extensively metabolized in the liver to the active metabolite GS-461203 and then dephosphorylated to the inactive substance GS-31007 [17,29,30].

##### Effects of Velpatasvir/Sofosbuvir on Other Drugs

Velpatasvir is a weak inhibitor of P-glycoprotein, BCRP, OATP1B1, and OATP1B3. Its effects on other drugs are limited to the absorption process. Co-administration with drugs that are substrates of these transporters may potentially increase their plasma concentrations.

Sofosbuvir and GS-331007 do not affect CYP enzymes or drug transporters. Therefore, they are not responsible for drug interactions, including with medications used in psychiatry [17,18,27].

##### Effects of Other Drugs on Velpatasvir/Sofosbuvir

Velpatasvir and sofosbuvir (but not GS-331007) are substrates of P-glycoprotein and BCRP. Velpatasvir is also a substrate of OATP1B, CYP2B6, CYP2C8, and CYP3A4 and is characterized by slow metabolism. Therefore, drugs that are strong inducers of P-gp or these CYP isoenzymes (e.g., St. John’s wort, carbamazepine, or phenobarbital) may reduce plasma concentrations of SOF and VEL, leading to reduced therapeutic effect. The use of these substances with velpatasvir/sofosbuvir is contraindicated. Concomitant use of drugs that are moderate P-gp or CYP inducers (e.g., oxcarbazepine or modafinil) is not recommended because they may reduce the plasma concentrations of both sofosbuvir and velpatasvir, thereby reducing the effectiveness of HCV treatment [17,18,29,30].

Although co-administration with medicinal products that inhibit P-gp or BCRP may increase SOF or VEL concentrations, and drugs that inhibit OATP, CYP2B6, CYP2C8, or CYP3A4 may increase velpatasvir plasma concentrations, this does not result in clinically significant drug interactions (e.g., methadone). The VEL/SOF regimen can be coadministered with P-gp, BCRP, OATP, and CYP inhibitors [17,18].

#### 3.1.3. Voxilaprevir

Voxilaprevir is an NS3/4A protease inhibitor that, along with velpatasvir and sofosbuvir, is part of a pangenotypic regimen used to treat HCV infections that have not responded to previous DAA therapy.

##### Effects of Voxilaprevir on Other Drugs

Voxilaprevir is an inhibitor of P-glycoprotein, BCRP, and OATP1B1/3. Therefore, co-administration with substrates of these transporters may lead to clinically significant increases in plasma concentrations of these drugs. Monitoring or dose adjustment of these drugs may be required when used concomitantly with voxilaprevir [19,27].

##### Effects of Other Drugs on Voxilaprevir

Voxilaprevir, like velpatasvir and sofosbuvir, is a substrate of the drug transporters P-gp and BCRP. It is also a substrate of OATP1B1 and OATP1B3. It is metabolized primarily in the liver by CYP3A4. Concomitant use of strong P-gp and CYP inducers with VOX is contraindicated, while concomitant use of moderate inducers is not recommended [19,27].

Drugs that are strong OATP1B inhibitors may significantly increase voxilaprevir plasma concentrations. However, the safety significance of this has not been determined.

The VOX/VEL/SOF regimen can be used concomitantly with P-gp, BCRP, and CYP inhibitors, as a clinically significant increase in plasma concentrations of these drugs is not expected [19].

### 3.2. Effect of DAAs on Psychotropic Drugs

Benzodiazepines and antidepressants are the most commonly used psychotropic drugs. They are extensively metabolized by CYP enzymes; for example, most benzodiazepines are substrates of CYP3A4, CYP2B6, and CYP1A2, which are inhibited by DAAs. These psychoactive drugs have a narrow therapeutic range and a strong correlation between drug concentration and effect [31]. Drug interactions between DAAs and benzodiazepines can lead to increased plasma concentrations of psychoactive substances and an increased likelihood of clinically significant toxicity [4,32]. Midazolam, a model CYP3A4 substrate, is one of the most extensively studied drugs in this context. Studies have shown a significant increase in its plasma concentration with the use of earlier protease inhibitors, such as boceprevir, paritaprevir/ritonavir, ombitasvir, and the NS5B inhibitor dasabuvir. The combination of these drugs with midazolam was contraindicated [5]. The currently used pangenotypic GLE/PIB regimen increases the area under the concentration–time curve (AUC) of midazolam by 27% after a single 1 mg dose, which is not clinically significant and does not require dosage adjustment. The other two pangenotypic regimens, VEL/SOF and VOX/VEL/SOF, do not affect the concentration of this drug [17,19].

Selective serotonin reuptake inhibitors are metabolized in the liver by CYP isoenzymes, primarily CYP3A4, but also CYP2D6, CYP2C9, and CYP2C19. These drugs have a broad therapeutic window; therefore, the inhibitory effect of some DAAs on CYP is not clinically significant [11].

Serotonin–norepinephrine reuptake inhibitors used with older genotype-specific DAA regimens that inhibit CYP3A4 sometimes require dose adjustments of the psychotropic substances. Currently used pangenotypic regimens have no or negligible effect on this inhibitor’s metabolism [33].

Antipsychotics/neuroleptics (aripiprazole, quetiapine, olanzapine, clozapine, risperidone) are metabolized in the liver by various CYPs, e.g., CYP3A4, CYP1A2, and CYP2D6, which can be inhibited by DAAs. Most antipsychotics have a narrow therapeutic index, and drug interactions with DAAs may alter the pharmacological effects of these psychoactive substances. The currently used GLE/PIB regimen, although weakly inhibiting CYPs, may affect the concentration of many neuroleptics, and close monitoring of patients for toxic effects is recommended (aripiprazole, quetiapine, clozapine). An exception is olanzapine, metabolized by CYP1A2, where glecaprevir/pibrentasvir has a minor effect, and the change in psychoactive drug concentration is not clinically significant. The other two DAA regimens, VEL/SOF and VOX/VEL/SOF, do not significantly affect antipsychotic drug levels [5,16,17,19,28].

Psychotropic medications are rarely responsible for DDIs. Some, such as benzodiazepines, have limited effects on CYP enzymes and transporter proteins, while others, such as antipsychotics, SSRIs, and tricyclic antidepressants, inhibit CYP2D6. However, this does not significantly affect the concentration of pangenotypic DAA regimens [5].

Neuropsychiatric medications belonging to the first-generation anticonvulsants, such as phenobarbital, phenytoin, carbamazepine, oxcarbazepine, and primidone, are another concern. These medications strongly induce P-gp and/or CYP3A4, CYP2B6, and CYP2C8, significantly reducing DAA exposure and, consequently, reducing antiviral efficacy. All of these medications are contraindicated or not recommended in combination with drugs that directly act on HCV [22,27]. There are a few reports of a small number of patients who remained on carbamazepine despite initiation of DAAs and achieved a sustained virologic response [34]. It is believed that if anticonvulsant therapy is required, switching to levetiracetam is recommended after discontinuing other anticonvulsants at least 1 week before initiating anti-HCV therapy [35].

The metabolic pathways of psychotropic medications and their potential interactions with pangenotypic DAA regimens are summarized in Table 1. Most of the interactions described have not been studied but are based on metabolism and clearance.

## 4. Summary

Chronic HCV infection affects nearly 50 million people [65]. The prevalence of this infection is higher among people with psychiatric disorders than in the general population. A particularly high prevalence, reaching 60–70%, is observed in those addicted to psychoactive substances administered intravenously [2,3]. In the past, due to numerous psychiatric side effects of interferon-based therapy used to treat HCV infection, these patients often could not be treated or required particularly close supervision [66]. The introduction of interferon-free DAA regimens has eliminated mental illness as a contraindication for HCV treatment [67]. However, potential DDIs with psychotropic medications remain a significant clinical challenge [24,25]. A 2010 study conducted by American insurers indicated that antidepressants were among the four most frequently used classes of medications in patients with chronic hepatitis C [68].

The retrospective studies by Turnes et al. concerning, among others, drugs used in mental disorders and pangenotypic DAA indicated a lower incidence of adverse events related to DDI when a regimen without a protease inhibitor was used [24,25]. Similar observations were made by Sicras-Mainar et al. in patients using cardiovascular therapies [26].

All currently available pangenotypic therapies interact with certain anticonvulsants. Finding a suitable alternative that does not involve DDIs while still providing patient protection can be difficult. If switching medications is not possible, careful monitoring is necessary [34].

Introducing DAAs to patients receiving pharmacotherapy for mental disorders also presents a complex challenge. Drug interactions can lead to toxic symptoms or exacerbation of the mental illness, further complicating antiviral therapy [25]. If the current treatment for a psychiatric disorder is effective, switching is often not a viable option. In such a situation, before initiating DAAs, the potential interactions with the patient’s medications should be carefully analyzed, and a treatment regimen for HCV infection with fewer DDIs should be selected [69].

A strategy for avoiding significant drug interactions requires a detailed medication history, including over-the-counter medications and herbal remedies (e.g., St. John’s wort), before and during DAA therapy. Furthermore, for psychiatric conditions treated with medications that interact with the proposed DAA regimens, discussion within interdisciplinary teams, including a psychiatrist and the physician treating HCV infection, is essential to determine appropriate management. This strategy should include a change in psychiatric management (drug change, dose adjustment, close monitoring for toxicity and/or psychiatric exacerbation) or selecting a different DAA regimen if these options are not feasible. Online resources, such as the University of Liverpool’s Hep Drug Interactions database (www.hep-druginteractions.org, accessed on 16 December 2025), can assist clinicians in assessing DDIs. This website offers DDI searches, an explanation of their mechanisms, and recommendations for further management. The 2018 recommendations of the European Association for the Study of the Liver on the treatment of chronic hepatitis C are also a valuable source of information, as they also emphasize the problem of DDI [27].

It is important to remember that most potential drug interactions have not been studied in humans, and recommendations are based on theoretical interpretations of drug pharmacokinetic characteristics [5].

## Figures and Tables

**Table 1:** Psychotropic agents’ route of metabolism and potential drug interactions.

Drug [References]	Route of Metabolism	Potential Interactions with DAA					Comments
		GLE	PIB	VOX	VEL	SOF	
**Benzodiazepines**							
Alprazolam [28,36]	Substrate CYP3A4	-	-	-	-	-	No interaction expected. GLE/PIB is a weak inhibitor of CYP3A4 and is unlikely to affect the exposure of alprazolam.
Bromazepam [28,37]	Substrate CYP1A2, CYP2D6	-	-	-	-	-	No interaction expected.
Chlordiazepoxide [28,38]	Substrate CYP3A4	-	-	-	-	-	No interaction expected.
Diazepam [28,39]	Substrate CYP3A4, CYP2C19	-	-	-	-	-	No interaction expected. GLE/PIB is a weak inhibitor of CYP3A4 and is unlikely to affect the exposure of diazepam.
Lorazepam [5,28]	Substrate multiple UGTs	-	-	-	-	-	No interaction expected.
Midazolam [16,28]	Substrate CYP3A4, CYP2B6	-	-	-	-	-	Co-administration with GLE/PIB increased midazolam exposure, but it is not clinically significant, and dose adjustment is not required.
Oxazepam [28]	Substrate UGT	-	-	-	-	-	No interaction expected.
Zolpidem [28,40]	Substrate CYP3A4, CYP1A2, CYP2C19	-	-	-	-	-	No interaction expected.
Zopiclone [28,41]	Substrate CYP3A4, CYP2C8	-	-	-	-	-	No interaction expected.
**SSRIs**							
Citalopram [28,42]	Substrate CYP3A4, CYP2C19, CYP2D6 Inhibitor weak: CYP2D6, CYP2C19, CYP1A2, CYP2B6	-	-	-	-	-	No interaction expected.
Duloxetine [28,43]	Substrate CYP1A2, CYP2D6, Inhibitor moderate: CYP2D6	-	-	-	-	-	No interaction expected.
Escitalopram [28,42]	Substrate CYP3A4, CYP2C19, Inhibitor weak: CYP2D6	-	-	-	-	-	No interaction expected.
Fluoxetine [28,44]	Substrate CYP2C9, CYP2D6 Inhibitor: Strong: CYP2D6, moderate: CYP2C19, Weak: CYP1A2, CYP2B6, CYP2C9	-	-	-	-	-	No interaction expected.
Paroxetine [28,45]	Substrate CYP2D6 Inhibitor strong: CYP2D6, moderate: CYP2B6, weak: CYP2C19, CYP2C9, CYP1A2	-	-	-	-	-	No interaction expected.
Sertraline [28,46]	Substrate CYP2C19, CYP3A4, CYP2B6, CYP2D6, CYP2C9 Inhibitor moderate: CYP2B6, CYP2C19, CYP2D6, weak: CYP1A2, CYP2C8, CYP2C9	-	-	-	-	-	No interaction expected.
Trazodone [28,47,48]	Substrate CYP3A4 Inducer: P-gp	-	-	-	-	-	No interaction expected.
Venlafaxine [28,49,50]	Substrate CYP2D6, CYP3A4 Inhibitor weak: CYP2B6, CYP2D6, CYP3A4	-	-	-	-	-	No interaction expected.
**Tricyclic antidepressants**							
Amitriptyline [28,51]	Substrate CYP2D6, CYP2C19, P-gp Inhibitor weak: CYP1A2, CYP2C19, CYP2C9, CYP2D6 CYP2E1	-	-	-	-	-	No interaction expected.
Doxepin [28,52]	Substrate CYP2D6, CYP2C19, CYP1A2, CYP2C9	-	-	-	-	-	No interaction expected
Imipramine [5,28,53]	Substrate CYP2C19, CYP2D6, CYP1A2, CYP3A4 Inhibitor moderate: CYP2D6, weak: CYP1A2, CYP2C19, CYP2E1	-	-	-	-	-	No interaction expected.
**Other antidepressants**							
Mianserin [28,54]	Substrate CYP2D6, CYP1A2, CYP3A4	-	-	-	-	-	Potential weak interaction with GLE/PIB.
Mirtazapine [5,28]	Substrate CYP1A2, CYP2D6, CYP3A4, CYP2C9 Inhibitor weak: CYP1A2	-	-	-	-	-	No interaction expected.
Moclobemide [5,28,55]	Substrate CYP2C19, CYP2D6 Inhibitor moderate: CYP2C19, weak: CYP1A2, CYP2D6	-	-	-	-	-	No interaction expected.
St John’s wort [28,56]	Inducer: CYP3A4, P-gp, CYP2B6, CYP2C8, CYP3A4, CYP1A2	↓	↓	↓	↓	↓	Do not coadminister. Co-administration with pangenotypic DAA may be considered if the total daily hyperforin dose is less than 1 mg.
**Antipsychotics**							
Aripiprazole [28,57]	Substrate CYP2D6, CYP3A4	-	-	-	-	-	Potential interaction with GLE/PIB. Aripiprazole has a narrow therapeutic index—monitor patients closely for signs and symptoms of toxicity when administered with GLE/PIB.
Clozapine [28,58]	Substrate CYP1A2, CYP3A4, CYP2C19, CYP2D6.	-	-	-	-	-	Potential interaction with GLE/PIB. A clinically significant interaction is unlikely. Close monitoring is recommended when administered with GLE/PIB.
Haloperidol [5,28,59]	Substrate CYP2D6, CYP3A4, UGT Inhibitor moderate: CYP2D6	-	-	-	-	-	No interaction expected.
Lurasidone [28,60]	Substrate CYP3A4, BCRP, P-gp Inhibitor weak: CYP3A4	-	-	-	-	-	Potential weak interaction with GLE/PIB. Exposure to Lurasidone may increase with GLE/PIB. No prior Lurasidone dose change is recommended.
Olanzapine [28,61]	Substrate CYP1A2, CYP2D6,	-	-	-	-	-	No interaction expected.
Paliperidone [28,62]	Substrate P-gp	-	-	-	-	-	Potential interaction with GLE/PIB, VOX, and VEL. A concentration of paliperidone may increase when administered with GLE/PIB, VOX, and VEL. A prior paliperidone dose change is not required. Close monitoring for side effects is recommended.
Quetiapine [28,63]	Substrate CYP3A4, CYP2D6	-	-	-	-	-	Potential interaction with GLE/PIB. Caution is advised when using higher doses of quetiapine (over 400 mg/day) with GLE/PIB.
Risperidone [28,64]	Substrate CYP2D6, CYP3A4, P-gp	-	-	-	-	-	Potential weak interaction with pangenotypic DAA.

BCRP—breast cancer resistance protein; CYP—cytochrome; GLE—glecaprevir; PIB—pibrentasvir; P-gp—P-glycoprotein; SOF—sofosbuvir; SSRIs—selective serotonin reuptake inhibitors; UGT—uridine diphosphate; VEL—velpatasvir; VOX—voxilaprevir.

## Data Availability

No new data were created or analyzed in this study.

## Abbreviations

The following abbreviations are used in this manuscript:

AUC	area under the concentration–time curve
BCRP	breast cancer resistance protein
CYP	cytochrome
DAAs	direct-acting antivirals
DDI	drug interactions
GLE	glecaprevir
HCV	chronic hepatitis C virus
OATP	organic anion-transporting polypeptide
P-gp	P-glycoprotein
PIB	pibrentasvir
SOF	sofosbuvir
SSRIs	selective serotonin reuptake inhibitors
UGT	uridine diphosphate
VEL	velpatasvir
VOX	voxilaprevir
